# Effect of Feeding Glycerin on Ruminal Environment and In Situ Degradability of Feedstuffs in Young Bulls

**DOI:** 10.3390/ani9060359

**Published:** 2019-06-16

**Authors:** Josefa Madrid, Silvia Martínez, Carmen Villodre, Miguel J. López, Juan Alcázar, Juan Orengo, Guillermo Ramis, Fuensanta Hernández

**Affiliations:** Department of Animal Production, Faculty of Veterinary, University of Murcia, Campus de Espinardo, 30100 Murcia, Spain; silviamm@um.es (S.M.); carmenvitu@gmail.com (C.V.); mjlopeza@um.es (M.J.L.); juan.alcazar@um.es (J.A.); jorengo@um.es (J.O.); guiramis@um.es (G.R.); nutri@um.es (F.H.)

**Keywords:** glycerol, beef cattle, ruminal metabolism, ruminal microorganism, ruminal degradation

## Abstract

**Simple Summary:**

Glycerin (a by-product of the biodiesel industry) can be used as an energetic ingredient in animal feeds, but there are few studies on fattening ruminants that concurrently analyse the effect of glycerin on the ruminal metabolism, ruminal bacterial population and feedstuff degradability. We have studied the effect of feeding glycerin in young bulls that were fed high levels of concentrate. The results showed that the inclusion of glycerin in beef cattle feed (at 20, 40 or 80 g/kg) increased the propionic acid production, and at high levels could reduce the ruminal pH. In addition, the ruminal population of *Selenomonas ruminantium* (a lactate- and glycerol-consuming and a propionate-producing species) could be increased by glycerin supplementation. In general, no negative effects on the ruminal degradability of feedstuffs (cereals, protein supplements and fibrous materials) are caused by the use of glycerin. In young bulls that were fed high levels of concentrate, glycerin at 20 or 40 g/kg of feed can be included without affecting the ruminal pH.

**Abstract:**

This work studied the effect of feeding glycerin in bulls that were fed high levels of concentrate on the ruminal environment and in situ degradability of feedstuffs. Four ruminally cannulated young bulls were assigned to a 4 × 4 Latin square arrangement of treatments. The diets consisted of 15% barley straw and 85% concentrate in dry matter (DM). There were four different concentrates: without glycerin, and with 20, 40 or 80 g of glycerin per kg of DM. Each diet was offered for 24 days, the ruminal fluid was sampled to evaluate the ruminal metabolism and to determine the ruminal bacterial population by quantitative real-time polymerase chain reaction analysis, and the in situ degradability of six different feedstuffs was measured. The treatment with the highest level of glycerin provided the lower pH (*p* < 0.001), and the acetic/propionic molar ratio decreased (*p* < 0.001) as glycerin increased. The incorporation of glycerin in the diet did not affect the DNA copies/µL of the total bacteria, *Butyrivibrio fibrisolvens* and *Butyrivibrio proteoclasticus* (*p* > 0.05) in the DNA extract of rumen fluid, but at high levels increased *Selenomonas ruminantium* (*p* < 0.01). Very few effects of glycerin incorporation were found for the in situ degradability. In young bulls that were fed high levels of concentrate, glycerin at 20 or 40 g/kg of feed could be included without affecting the ruminal pH and raising the propionic acid, but at 80 g/kg the ruminal pH dropped, despite the increase of *Selenomonas ruminantium*.

## 1. Introduction

The feasibility of using glycerin (a by-product of the biodiesel industry) as an energy ingredient in diets for both ruminants and non-ruminants has been confirmed [1]. Glycerol, the major component of glycerin, generates glucose through the action of the liver, becoming an energy substrate for the cellular metabolism. In ruminants, the glycerol is extensively fermented in the rumen into propionic and butyric acids [2,3], propionic acid being a metabolic precursor of glucose [4]. The incorporation of glycerin improves the efficient use of the feed, due to a decrease in the acetic/propionic ratio in the rumen [5]. Whitney et al. [6] showed a higher growth in beef heifers related to an increase in blood glucose, linked with a greater production of propionic acid in the rumen medium. Some studies have observed that the inclusion of glycerin in moderate amounts can improve the feed conversion ratio in growing or finishing ruminants [7,8], but that higher amounts may even have undesirable effects on this parameter [8]. These facts could be closely related to the effect of the addition of glycerin on the ruminal metabolism.

Recently, a potential effect in preventing acidosis has been indicated for glycerin, due to the promoting effect of glycerol on lactate-using bacteria such as *Megasphaera elsdenii* [9], but there are few studies on fattening ruminants that extensively analyse the effect of glycerin on the ruminal metabolism, ruminal population and feedstuff degradability. As a consequence, the hypothesis of this work was that the ruminal fermentation of glycerin could benefit some microorganisms, impeding negative effects on the ruminal metabolism and on the degradability of dietary ingredients. The aim of this work was to study the effect of feeding glycerin in young bulls that were fed high levels of concentrate on the ruminal environment and the in situ degradability of feedstuffs.

## 2. Materials and Methods

The Ethics Committee of the University of Murcia and the Administrative Authorities (Murcia, Spain) approved this protocol (A13171003), and all of the experimental procedures were in compliance with the European Union regulations concerning the protection of animals used for scientific purposes (2010/63/EU Directive).

### 2.1. Animal and Experimental Design

The study was conducted over 96 days at the Experimental Unit of Animal Nutrition (University of Murcia, Spain). Four ruminally cannulated young bulls (Limousin x indigenous Spanish breed) of 292.8 ± 29.6 kg of BW of eight months of age were used in the experiment. The young bulls were assigned to a 4 × 4 Latin square arrangement of treatments; four diets were evaluated, and each animal received an experimental diet for a 24-day period (14 days of adaptation and 10 days of sample collecting); all the animals received the 4 diets, and each permutation of the diets was set as a rotation. The young bulls were housed in individual pens (5 m × 4 m) with a straw-covered floor, provided with feeders, and with free access to water.

The diets consisted of 15% barley straw and 85% concentrate in dry matter (DM). There were four different concentrates: without glycerin, and with 20, 40 or 80 g of glycerin per kg of DM (0 G, 20 G, 40 G and 80 G, respectively) (Table 1). The glycerin was incorporated to partially replace the feed barley. The metabolizable energy of glycerin was estimated according to the Fundación Española para el Desarrollo de la Nutrición Animal (FEDNA) [10]. In addition, the diets were formulated to meet or exceed the requirements of young bulls, as indicated by FEDNA [11], and to be iso-nitrogenous and iso-energetic.

During the adaptation period, straw was offered ad libitum, and the concentrate was offered in a mash twice daily at 08:00 h and 17:00 h, allowing 10% orts. The amount of concentrate offered was adjusted to fulfil the target forage/concentrate ratio. On day 16, the young bulls were restricted to 90% of the intake determined in the adaptation period (5.91 kg of DM/day), to ensure the absence of orts during the sampling period.

### 2.2. Sampling of Ruminal Fluid and Fermentation

The ruminal fluid was sampled 0, 2, 4 and 8 h after the morning feeding over two consecutive days, day 17 and day 18 of the period. The sample digesta (approximately 500 mL) were collected from at least 4 locations (anterior dorsal, anterior ventral, posterior dorsal and posterior ventral) within the rumen. The ruminal pH was measured immediately using an electric pH meter (pH meter Crison GLP 21+, Crison Instruments, S.A., Alella, Barcelona, Spain). After that, the samples were strained through four layers of cheesecloth. 2 mL subsamples from 0 and 8 h of day 17 were immediately frozen at −80 °C until quantitative real-time polymerase chain reaction (qPCR) analysis. Subsequently, the filtrates from both days and all the sampling times were centrifuged at 3000× *g* for 20 min. The subsamples (8 mL) of each fluid were stored at −20 °C to analyse the lactic acid and ammonia concentrations. In addition, other 10 mL subsamples were transferred into plastic containers, and 0.2 mL of 50% sulphuric acid was added, before freezing at −20 °C until the volatile fatty acids (VFA) determination.

The lactic acid and ammonia concentrations were analysed spectrophotometrically, as described by Taylor [12] and Chaney and Marbach [13], respectively. The VFA concentration was measured by capillary gas chromatography, as described by Madrid et al. [14]. The gas chromatograph (TRACE GC Ultra, Thermo Finnigan Italia SpA, Milan, Italy) was equipped with a flame ionisation detector. The capillary column was fused silica, 30 m × 0.25 mm × 0.25 μm ID, and coated with FFAP-TR as the stationary phase (Teknokroma, Barcelona, Spain). Standard solutions of acetic, propionic, butyric, isobutyric and isovaleric acids were prepared for calibration, using 4-methyl-n-valeric acid (30 mmol/L) as the internal standard. Original solutions were diluted twice, and the internal standard was added, at a ratio of 10:1 (diluted supernatant/internal standard). The solution was mixed and centrifuged at 7000× *g* for 7 min at 4 °C. Then, a 1 μL aliquot of this solution was injected onto the column.

### 2.3. DNA Extraction

Bacterial DNA was extracted from 200 µL of rumen fluid using the Danagene Spin Genomic DNA Kit (Danagene Bioted, Barcelona, Spain), according to the manufacturer’s instructions. The quantity of DNA was assessed using a Nanodrop ND-1000 spectrophotometer (Thermo Fisher Scientific Inc, Wilmington, DC, USA).

### 2.4. Quantitative Real-Time PCR

The primers for detecting the ruminal bacteria 16S rRNA gene sequences were previously used by Abo El-Nor et al. [15]. The primer pair sequences (5′–3′) and the annealing temperatures used to analyse the four target bacteria were: total bacteria (forward CGGCAACGAGCGCAACCC and reverse CCATTGTAGCACGTGTGTAGCC; 60 °C); *Butyrivibrio fibrisolvens* (forward TAACATGAGTTTGATCCTGGCTC and reverse CGTTACTCACCCGTCCGC; 62 °C); *Butyrivibrio proteoclasticus* (previously named *Clostridium proteoclasticum*) (forward TCCTAGTGTAGCGGTGAAATG and reverse TTAGCGACGGCACTGAATGCCTAT; 62 °C); and *Selenomonas ruminantium* (forward TGCTAATACCGAATGTTG and reverse TCCTGCACTCAAGAAAGA; 54 °C).

All primers were purchased from TIB MOLBIOL GmbH (Berlin, Germany). The qPCR analyses were performed in a 7300 ABI thermocycler with a 7500 Fast System Software Version 1.4.0. (Applied Biosystems, Foster City, CA, USA). Each reaction was run in a total volume of 25 µL, which contained 12.5 µL GoTaq^®^ qPCR Master Mix (Promega, Madison, WI, USA), 0.25 µL of CXR reference dye (Promega, Madison, USA), 0.125 µL of each primer (50 nM), 11 µL of RNAse free water and 1 µL of DNA extract (100 ng/µL). The cycling conditions were as follows: one denaturation cycle at 95 °C for 10 min, followed by forty cycles of 30 s denaturation at 95 °C, 30 s annealing and a 1 min extension at 72 °C. The fluorescence data was obtained at the extension step. The melting curves were checked to verify the amplification of the expected PCR product. The qPCR efficiency was 1.95 (88%), as offered by the thermal cycler software. The quantification was carried out using standard curves generated by 10-fold serial dilutions of the purified PCR products with a known bacterial DNA concentration.

### 2.5. In Situ Degradability of Substrates

The ruminal degradation kinetics of corn, barley, soybean meal, corn distillers dried grains with solubles (DDGS), barley straw and sugar beet pulp were measured using the nylon bag technique. The chemical composition of the substrates is presented in Table 2. All of the substrates were oven-dried at 60 °C for 48 h, and were then ground (Retsch ZM 200 Ultra Centrifugal Mill; Retsch, Haan, Germany) to pass a 2 mm screen. About 3 g of barley straw and sugar beet pulp were weighed into 10 cm × 15 cm nylon bags, and 1.5 g of concentrates were introduced into a 5 cm × 10 cm nylon bag. All of the nylon bags had a pore size of 50 µm (Ankom Technology, Macedon, NY, USA), and after being filled they were thermally sealed. Two bags per substrate, time and treatment were prepared. In addition, two empty bags (blanks) were included for each time and treatment.

The bags were introduced through the cannula into the rumen of each young bull, and were incubated for 0, 2, 4, 8, 16, 24 and 48 h for the concentrates, and plus 72 h for barley straw and sugar beet pulp. Two sets of in situ incubations were performed per animal and period. The first incubation of 48 h for concentrate feedstuffs was performed on days 20 and 21, and the second incubation of 72 h for fibrous substrates was performed on days 22, 23 and 24 of each period.

After removal, the nylon bags were immediately introduced into an ice box and taken to the laboratory, where they were washed in three consecutive washing cycles with clean and cold water, each lasting 5 min, using a mechanical washer without centrifugation. After washing, the samples were dried in a forced-air oven at 60 °C until constant weight, and then weighed to determine the degradability of the dry matter of the materials. The relationship between the disappearance of the substrate dry matter from the nylon bags and the incubation time (t) was analysed using the non-linear equation procedure of the IBM SPSS software, according to the following exponential equation [16]:

(1) $$D\left(t\right)=a+b\;\left(1-e^{-ct}\right)$$

where D(t) is the proportion (%) of dry matter at time t (hours of incubation); a is the rapidly soluble fraction (%); b is the potentially degradable fraction (%); and c is the rate of degradation (h^−1^) of the fraction b. The potential degradability values (PD, %) were estimated as the sum of a+b, and the effective degradability values (*ED*, %) were estimated as $ED\;=a+\left[\frac{bc}{c+k}\right]$, where a, b, and c are the estimated degradation constants, and k is the fractional rate of passage assumed to be 0.06 h^−1^.

### 2.6. Diet and Feedstuff Analysis

The samples from the diets and feedstuffs used for the degradability test were ground to pass a 1 mm screen (Retsch ZM 200 Ultra Centrifugal Mill; Retsch, Haan, Germany) for a chemical analysis. The DM content of the samples was determined by drying in a convection oven at 105 °C for 8 h. The diets were analysed for crude protein (CP) and ash by the Association of Official Analytical Chemists procedures [17]; neutral detergent fiber (NDF) and acid detergent fiber (ADF) by the Van Soest et al. [18] methods; and starch by the official analytical method described in the Boletín Oficial del Estado [19]. The glycerol content of the diets was analysed by gas chromatography, as described by Madrid et al. [20]. In the feedstuffs used for the degradability test, acid detergent lignin (ADL) was determined through the solubilization of cellulose with 72% sulphuric acid, and the ether extract was analysed according to AOAC [17]; and CP, ash, NDF and ADF were analyzed in the same way as in the diets.

### 2.7. Statistical Analyses

The average of the pH, ammonia, lactic acid and VFA concentrations for each sampling hour (across days 17 and 18) were used to represent the post-feeding fermentation of each period. For the DNA copy number, the values were logarithmically transformed (log10 DNA copy number) to meet a normal distribution before the statistical analysis. These measurements were analysed using the Mixed Procedure of IBM SPSS Statistics (IBM Corporation, Armonk, NY, USA). The statistical model used was as follows:

(2) $$\Upsilon_{ijklm}=\mu +G_{i}+T_{j}+GT_{ij}+A_{k}+P_{l}+\varepsilon_{ijklm}$$

where $\Upsilon_{ijklm}$ is the observed measurement, μ is the overall population mean, $G_{i}$ is the fixed effect of the diet treatment, $T_{j}$ is the fixed effect of the post-feeding hour analysed as a repeated measure, $GT_{ij}$ is the fixed effect of the interaction $G_{i}$ and $T_{j}$, $A_{k}$ is the random effect of the animal, $P_{l}$ is the random effect of the rotation and *ε_ijklm_* is the residual error. Additionally, another statistical model was applied to analyse the degradability data, with the diet treatment ($G_{i}$) as the fixed effect, and the animal ($A_{k}$) and rotation ($P_{l}$) as the random effects:

(3) $$\Upsilon_{iklj}=\mu +G_{i}+A_{k}+P_{l}+\varepsilon_{iklj}$$

The differences among the means were further compared using the LSD method. The experimental unit was the animal, and the significance level was set at *p* < 0.05.

## 3. Results

### 3.1. Diets and Ruminal Environment

The chemical composition of the rations showed that the incorporation of glycerin in the concentrate gave glycerol levels of 15.1, 35.1 and 69.8 g/kg of the DM in the 20 G, 40 G and 80 G dietary treatments, respectively (Table 1). Table 3 presents the effects of the glycerin inclusion on the ruminal environment of young bulls. There was a significant (*p* < 0.001) effect of the glycerin administration on the ruminal pH. The dietary treatment with the highest level of glycerin (80 G), yielded a lower pH value (<6.0) than the 0 G, 20 G and 40 G treatments (>6.0). The sampling time (*p* < 0.001) had a significant effect on the ruminal pH, the highest values were obtained at 0 h (6.73), and the lowest levels were reached at 4 h post-feeding (5.74), followed by the values at 2 and 8 h (6.03 and 6.09, respectively). There was no interaction (*p* > 0.05) between the treatment and post-feeding time.

Regarding the ammonia-N values in the rumen, significant differences (*p* < 0.05) were found between treatments, the 20 G diet providing the lowest concentration compared with the rest of the treatments. The effect of the sampling time on the rumen influenced the NH_3_-N level (*p* < 0.001), increasing in the first two hours (after feeding) before decreasing (>4 h after feeding). An interaction between the diet and time was not found (*p* > 0.05) for this parameter.

The lactic acid concentration was only affected by the sampling time (*p* < 0.05), reaching lower values at 4 h after feeding with respect to 0 and 2 h. The total VFA production was affected by the treatment (*p* < 0.01); therefore, the lowest levels were reached with 80 G, regardless of the post-feeding time. There was a significant effect of the sampling time (*p* < 0.01) on the total VFA concentration, reaching the highest levels from 2 h to 8 h after feeding. There was no interaction (*p* > 0.05) between the treatment and time on the total VFA.

The molar proportions of all VFA were affected by the treatment (*p* < 0.001), except for isobutyric acid (*p* > 0.05). The molar proportion of acetic acid diminished when the proportion of glycerin was increased in the diet, an effect that was particularly evident in the 40 G treatment, and even more in 80 G. In addition, the molar proportion of propionic acid was higher in the 20 G, 40 G and 80 G treatments. Thus, the acetic/propionic molar ratio decreased as glycerin increased, especially in the 40 G and 80 G treatments. Regarding the molar proportion of butyric acid, the 20 G and 40 G treatments showed lower levels than the control, while the 80 G increased the values of this acid in the rumen. However, the ratio of (acetic + butyric)/propionic decreased as the proportion of glycerin increased. For the case of the molar percentage of isovaleric acid, its proportion decreased to a similar extent in the 20 G, 40 G and 80 G treatments with respect to the control diet; and for valeric acid, its proportion was higher in the 40 G and 80 G.

The effect of time after feeding on the ruminal molar proportions of VFA showed differences for all acids (*p* < 0.05), except for isovaleric acid (*p* > 0.05), while no interactions between the diets and post-feeding times were found. The acetic acid proportion was lower at 2, 4 and 8 h than at 0 h after feeding, although at 8 h it was higher than at 4 h. Propionic acid was higher for all of the post-feeding times than at 0 h. Therefore, the acetic/propionic and (acetic + butyric)/propionic ratios decreased after feeding. The butyric acid and valeric acid proportions were higher at 2 and 4 h than at 0 h after feeding, although at 8 h intermediate values were found for both VFA. The proportion of isobutyric acid was lower at 2, 4 and 8 h than at 0 h.

### 3.2. Microbial Population

The incorporation of glycerin in the diet did not affect (*p* > 0.05) the studied microbial indicators (Table 4), with the exception of *Selenomonas ruminantium* (*p* < 0.01). This microorganism increased substantially when high levels of glycerin (80 G) were incorporated. No effects of the time after feeding (0 and 8 h), or of the interactions between the diet and time were found (*p* > 0.05).

### 3.3. In Situ Degradability

The in situ DM degradability of cereals (corn and barley grains) and soybean meal was not affected (*p* > 0.05) by the inclusion of glycerin (Table 5). In contrast, for the ruminal degradation kinetics of DDGS, the rapidly soluble fraction (*a*) for 80 G was lower (*p* < 0.05) than for the 0 G and 40 G treatments; however, the rate of degradation of the fraction *b* (*c*) was the highest (*p* < 0.05) for 80 G. These contrary effects meant that when the effective degradability was estimated, there were no significant differences (*p* > 0.05) between the treatments.

In addition, no significant differences were found (*p* > 0.05) between the treatments, as regards the degradability parameters and effective degradability for two fibrous materials (barley straw and sugar beet pulp). However, the rapidly soluble fraction (*a*) of straw was slightly higher (*p* < 0.01) in the 80 G treatment.

## 4. Discussion

In our trial, the 80 G treatment resulted in the lowest pH value (5.74). Similar results were found by Mach et al. [21] in Holstein bulls that were fed high-concentrate diets, in which the administration of a concentrate with 8% glycerin resulted in the lowest ruminal pH value (5.68), although feeds with 0, 4 or 12% of glycerin reached higher ruminal pH values. These authors explained this by the observation that the diet with 8% glycerin was the one that was consumed at the highest quantity, which could affect the ruminal pH. Nagaraja and Lechtenberg [22] indicated that a ruminal pH lower than 5.6 has a significant impact on the rumen function, setting that a pH value between 5.0–5.5 is a clinical sign of subacute acidosis. The ruminal pH values recorded in our experiment were close to the upper limit of this range, even though they did not exceed it. However, in our trial the diets were restricted to keep the forage/concentrate ratio constant. Kijora et al. [2] found that the ruminal pH was reduced to 5.4 when 200 g of glycerol (twice daily) was infused into the rumen in bulls that were fed a grain-based diet.

The 20 G diet yielded the lowest concentration of NH_3_-N in our experiment. In lactating cows, Shin et al. [23] observed a reduction of NH_3_-N in ruminal fluid when glycerol was included in the diet, possibly due to an improvement in the microbial utilization of N. However, this effect was not observed with higher levels of glycerin (4 and 8% glycerin feed).

In the present study, the 80 G diet decreased the total VFA levels. Hales et al. [24] indicated that the effect of glycerin inclusion on the rumen parameters was similar to nutrients like starch, and no effects were found on the total VFA level. Werner Omazic et al. [25] reported that not all glycerol is fermented in the rumen, since a considerable part could be absorbed directly, while another part could escape from the rumen; therefore, it is possible that, in our study, this fact affected the total VFA when glycerin replaced barley grain at a high level.

Regarding the proportion of ruminal VFA, the propionic acid molar percentage increased and the acetic/propionic ratio decreased when glycerin was included. This effect was expected, since several studies have indicated that the inclusion of glycerin in the diet increases the ruminal propionic acid values [23,24]. Furthermore, the effect of glycerin on the acetic/propionic ratio has been previously described [3,23,24,26,27]. It is known that propionic production is mainly due to the fermentation of non-structural carbohydrates by amylolytic bacteria. In addition, our study showed that the incorporation of glycerin increased the propionic acid production even more. The diets that were studied followed a post-feeding fermentation pattern that favored the production of propionic acid, due to the high-concentrate content, regardless of the level of incorporated glycerin. Several experiments have found that increasing the proportion of glycerin increases the butyric acid concentration in the rumen [3,23], although others have reported just the opposite or no effect [21,27]; but we found that the proportion of butyric acid depended on the level of glycerin incorporation.

The microbial population present in the rumen is responsible for the production of VFA, and any modification could affect the amount and/or proportion of VFA [23]. In our case, the total bacteria were constant for all of the treatments, and only the 8% glycerin feed addition decreased the VFA production slightly. Regarding a specific microorganism such as *Butyrivibrio fibrisolvens*, this was not affected by the treatments. This microorganism is a cellulolytic and xylanolytic rumen bacterium [28] with a proteolytic activity [29] and is an important producer of butyric acid. Additionally, McCain et al. [30] found no effects resulting from including glycerin in the feed of beef steers on the population of *Butyrivibrio fibrisolvens* when up to 12% glycerin was included in a high-forage diet. However, in vitro, it has been observed that glycerol at high levels can decrease the population of *Butyrivibrio fibrisolvens* [31], possibly because it impedes the access of the bacteria to nutrients.

Another studied microorganism that was not modified by the incorporation of glycerin was *Butyrivibrio proteoclasticus*. Moon et al. [32] indicated that this bacterium is able to use a carbon source, such as inulin, pectin, starch and xylan, and to produce VFA; furthermore, it has a proteinase activity. By contrast, in an in vitro study, Abo El-Nor et al. [15] found, using continuous fermenters, that the population of *Butyrivibrio proteoclasticus* was lower with glycerol than without glycerol diets.

In our trial, *Selenomonas ruminantium* increased substantially with the incorporation of high levels of glycerin, possibly because glycerol stimulates this microorganism. This bacterium can use monosaccharides and disaccharides as substrates, but also lactate and glycerol; in addition, it is a propionate-producing species [29]. Thus, we observed an increase in the molar proportion of propionic acid and a decrease in the acetic/propionic ratio when glycerin was added, especially at high levels. In beef steers, McCain et al. [30] found that the population of *Selenomonas ruminantium* increased up to 21-fold in the rumen after a 12% glycerin supplementation. Fernando et al. [33] indicated that an increase in *Selenomonas ruminantium* populations together with other species such as *Megasphaera elsdenii* could decrease the amount of lactic acid in high-concentrate diets, since these microorganisms are considered lactate-using species. Castagnino et al. [9] noted, despite not finding an increase in the population of *Selenomonas ruminantium*, that when adding 10% glycerin to the diets of beef cattle, there was an increase in *Megasphaera elsdenii*; furthermore, they did not find changes in other populations such as fibrolytic bacteria (*Ruminococcus albus* and *Fibrobacter succinogenes*).

We found no effects of glycerin incorporation on the effective degradability of DM for cereals (feedstuffs rich in non-fibrous carbohydrates). In contrast, Wang et al. [3] indicated that the in situ ruminal DM degradation of a concentrate mix (with > 50% corn grain) improved when glycerin was included in the diet. For soybean meal or for DDGS (substrates with a high protein content), very few or no effects were detected on the ruminal degradation kinetics. Kijora et al. [2] indicated a decrease of the protein metabolism of rumen bacteria when a high amount of glycerol was infused into the rumen, noticing that the branched-chain VFA (isobutyric and isovaleric acids) concentration dropped in the rumen fluid. In our experiment, the molar proportion of isovaleric acid decreased when glycerin was added, but this effect was not significantly detected for isobutyric acid. Other studies showed that the apparent digestibility of the fibrous fractions was reduced when the level of glycerin in the diet increased [23]. In our case, no differences were found on the effective degradability for two very different fibrous raw materials (barley straw and sugar beet pulp), when glycerin was increased. In addition, Castagnino et al. [9] reported that high-concentrate diets with 10% glycerin that were fed to beef cattle did not affect the apparent digestibility of DM or NDF, nor the abundance of fibrolityc bacteria.

## 5. Conclusions

The incorporation of glycerin in rich concentrate diets decreases the acetic/propionic ratio due to an intense propionic fermentation, and, at high levels, it could reduce the ruminal pH. The total bacteria, *Butyrivibrio fibrisolvens* and *Butyrivibrio proteoclasticus* were not negatively affected by the glycerin addition, while *Selenomonas ruminantium* (a lactate- and glycerol-consuming and a propionate-producing species) increased. Moreover, the absence of any effect of glycerin incorporation on the degradability of a large group of feedstuffs could be related to what was observed in relation to the microbial population, where no negative effects were found. In conclusion, even though adding glycerin showed benefits on the propionic acid production and *Selenomonas ruminantium* population without impairing the degradability of dietary ingredients, the inclusion of 80 g/kg of feed could drop the ruminal pH close to the limit in bulls that were fed high-concentrate diets. However, further studies should be developed to explore the impact of using glycerin on the ruminal environment in commercial farm conditions.

## Figures and Tables

**Table 1:** Ingredients and composition of the experimental diets.

Item	Treatments ^1^			
	0 G	20 G	40 G	80 G
Ingredient, g/kg DM ^2^				
Concentrate				
Barley grain	363.0	342.6	323.0	303.5
Corn grain	255.0	255.0	255.0	239.7
Corn Gluten feed	136.0	136.0	136.0	130.9
Soybean meal 47% CP ^3^	36.6	40.4	44.6	53.3
Palm oil	24.2	23.4	22.1	20.7
Glycerin ^4^	0.0 (0)	17.0 (20)	34.0 (40)	68.0 (80)
Calcium carbonate	14.5	14.5	14.5	14.5
Urea	4.3	4.3	4.3	4.3
Salt	3.0	3.0	3.0	3.0
Sodium bicarbonate	3.8	3.8	3.8	3.8
Magnesium oxide	1.3	1.3	1.3	1.3
Vitamin-mineral premix ^5^	8.5	8.5	8.5	8.5
Forage				
Barley straw	150.0	150.0	150.0	150.0
Calculated composition ^6^				
CP (g/kg DM)	128.0	128.0	128.1	128.9
Metabolizable energy (MJ/kg DM)	11.0	11.0	11.0	11.0
Analysed composition (g/kg DM)				
Ash	70.1	68.8	68.3	64.3
CP	125.4	131.8	126.7	130.9
Starch	407.3	403.9	367.4	358.0
NDF ^7^	329.4	345.5	316.6	323.4
ADF ^8^	204.1	194.4	195.8	195.7
Glycerol	-	15.1	35.1	69.8

^1^ Treatments consisted of 15% barley straw and 85% concentrate in dry matter. There were four different concentrates: without glycerin, and with 20, 40 or 80 g of glycerin per kg of dry matter (0 G, 20 G, 40 G and 80 G, respectively). ^2^ DM = Dry Matter. ^3^ CP = Crude Protein. ^4^ Number between parentheses indicates g/kg of glycerin (DM) in concentrate. The analysed composition of glycerin was: glycerol, 875 g/kg; water, 78 g/kg; ash, 59 g/kg; chloride, 30 g/kg; sodium, 20 g/kg; and methanol, 0.5 g/kg. ^5^ Provided (per kg of concentrate): vitamin A, 6000 IU; vitamin D3, 800; vitamin E, 15 mg; zinc (zinc oxide), 40 mg; Cu (copper sulfate), 5 mg; Se (sodium selenite), 0.20 mg; I (potassium iodide), 0.50 mg; Co (cobalt carbonate), 0.20 mg. ^6^ CP and metabolizable energy were calculated according to FEDNA [10]. ^7^ NDF = Neutral Detergent Fiber. ^8^ ADF = Acid Detergent Fiber.

**Table 2:** The chemical composition of the substrates used in the degradability test.

Item	Corn Grain	Barley Grain	DDGS ^1^	Soybean Meal	Barley Straw	Sugar Beet Pulp
Dry matter (DM; g/kg)	867.0	889.1	896.5	882.5	918.5	902.5
Analysed composition (g/kg DM)						
Crude protein	86.4	118.0	278.3	498.5	39.1	100.8
Ether extract	39.4	18.7	95.9	20.9	16.3	11.6
Ash	19.0	24.7	57.4	70.2	74.5	67.6
Neutral detergent fiber	113.5	197.1	342.4	143.9	764.3	466.5
Acid detergent fiber	32.8	63.0	132.7	84.4	513.8	254.2
Acid detergent lignin	8.6	12.3	33.4	4.20	84.3	18.8

^1^ DDGS = corn distillers dried grains with solubles.

**Table 3:** Effect of feeding glycerin on the ruminal environment of young bulls fed a high level of concentrate.

Item	Treatments ^1^				Hours after Feeding				SEM ^2^	*p*-Value		
	0 G	20 G	40 G	80 G	0 h	2 h	4 h	8 h		Treatment (G)	Hours (T)	GxT
pH	6.32 ^ab^	6.38 ^a^	6.14 ^b^	5.74 ^c^	6.73 ^a^	6.03 ^b^	5.74 ^c^	6.09 ^b^	0.183	<0.001	<0.001	0.164
NH_3_-N (mg/100 mL)	3.39 ^a^	2.32 ^b^	3.51 ^a^	3.78 ^a^	3.82 ^b^	5.12 ^a^	2.08 ^c^	1.98 ^c^	0.385	0.019	<0.001	0.089
Lactic acid (mg/100 mL)	4.14	3.96	4.31	4.30	4.79 ^a^	4.52 ^a^	3.35 ^b^	4.04 ^ab^	0.402	0.919	0.012	0.388
Total VFA ^3^ (mM)	105.2 ^a^	99.5 ^a^	100.6 ^a^	83.6 ^b^	85.5 ^b^	103.1 ^a^	99.5 ^a^	100.7 ^a^	4.67	0.001	0.009	0.082
Molar proportion (%)												
Acetic acid	60.5 ^a^	61.0 ^a^	58.3 ^b^	52.8 ^c^	62.9 ^a^	56.6 ^bc^	55.3 ^c^	57.8 ^b^	2.11	<0.001	<0.001	0.554
Propionic acid	24.1 ^c^	26.7 ^b^	29.0 ^a^	29.7 ^a^	23.8 ^b^	28.4 ^a^	29.5 ^a^	27.6 ^a^	3.24	<0.001	<0.001	0.614
Butyric acid	10.6 ^b^	9.14 ^c^	8.69 ^c^	13.1 ^a^	9.15 ^b^	10.8 ^a^	11.0 ^a^	10.5 ^ab^	1.46	<0.001	0.027	0.630
Isobutyric acid	0.52	0.36	0.43	0.27	0.62 ^a^	0.36 ^b^	0.24 ^b^	0.35 ^b^	0.073	0.062	0.005	0.052
Valeric acid	1.94 ^c^	2.04 ^c^	2.67 ^b^	3.18 ^a^	1.88 ^c^	2.62 ^ab^	2.95 ^a^	2.39 ^bc^	0.408	<0.001	0.003	0.966
Isovaleric acid	2.22 ^a^	0.70 ^b^	0.80 ^b^	0.86 ^b^	1.50	1.11	0.80	1.17	0.381	<0.001	0.280	0.963
Acetatic/propionic	3.03 ^a^	2.55 ^b^	2.13 ^c^	1.96 ^c^	2.96 ^a^	2.20 ^b^	2.11 ^b^	2.40 ^b^	0.425	<0.001	<0.001	0.951
(Acetatic+butyric)/propionic	3.62 ^a^	2.93 ^b^	2.46 ^c^	2.44 ^c^	3.41 ^a^	2.64 ^b^	2.55 ^b^	2.86 ^b^	0.542	<0.001	0.003	0.948

^1^ Concentrate without glycerin, with 20, 40 or 80 g of glycerin per kg of dry matter (0 G, 20 G, 40 G and 80 G, respectively). ^2^ SEM = standard error of the mean (n = 4 per treatment × 4 per post-feeding time). ^3^ VFA = Volatile fatty acids. ^a–c^ Means in the same row with different superscripts (within treatments or hours after feeding) are significantly different (*p* < 0.05).

**Table 4:** The effect of feeding glycerin on the total bacteria, *Butyrivibrio fibrisolvens*, *Butyrivibrio proteoclasticus* (previously called *Clostridium proteoclasticum*), and *Selenomonas ruminantium* in the DNA extract of rumen fluid from young bulls that were fed a high level of concentrate.

DNA Copies/μL (log10)	Treatments ^1^				Hours Post-Feeding		SEM ^2^	*p*-Value		
	0 G	20 G	40 G	80 G	0	8		Treatment (G)	Hour (T)	GxT
Total bacteria	10.03	10.13	9.45	9.47	10.10	9.93	0.071	0.778	0.240	0.967
*Butyrivibrio fibrisolvens*	7.27	7.23	6.98	7.17	7.23	7.09	0.063	0.356	0.253	0.408
*Butyrivibrio proteoclasticus*	3.87	3.92	3.52	3.24	3.87	3.40	0.196	0.514	0.230	0.951
*Selenomonas ruminantium*	0.21 ^b^	1.24 ^b^	1.24 ^b^	2.53 ^a^	1.42	1.19	0.205	0.004	0.561	0.767

^1^ Concentrate without glycerin, with 20, 40 or 80 g of glycerin per kg of dry matter (0 G, 20 G, 40 G and 80 G, respectively). ^2^ SEM = standard error of the mean (n = 4 per treatment × 2 per post-feeding time). ^a,b^ Means in the same row with different superscripts are significantly different (*p* < 0.05).

**Table 5:** The parameters of degradability and the potential and effective degradability of dry matter of different substrates in young bulls that were fed a high level of concentrate.

Item	Treatments ^1^				SEM ^2^	*p*-Value
	0 G	20 G	40 G	80 G		
Corn grain ^3^						
*a* (%)	33.0	29.5	27.2	27.6	1.01	0.220
*b* (%)	64.3	67.5	70.8	70.3	1.20	0.236
*c* (h^−1^)	0.067	0.070	0.063	0.074	0.005	0.791
Potential degradability ^4^ (%)	97.4	97.0	98.0	97.9	0.76	0.858
Effective degradability ^5^ (%)	66.6	65.2	63.0	65.7	1.03	0.692
Barley grain						
*a* (%)	30.5	27.8	27.4	30.3	0.45	0.057
*b* (%)	58.7	62.4	61.5	60.7	0.64	0.237
*c* (h^−1^)	0.394	0.368	0.380	0.407	0.054	0.994
Potential degradability (%)	89.2	90.3	89.0	91.0	0.47	0.266
Effective degradability (%)	80.6	78.8	79.3	82.4	1.10	0.456
DDGS ^6^						
*a* (%) ^7^	**53.9 ^a^**	**52.4 ^ab^**	**53.3 ^a^**	**47.7 ^b^**	**0.71**	**0.037**
*b* (%)	34.7	29.0	27.7	28.6	1.92	0.624
*c* (h^−1^)	**0.032 ^b^**	**0.032 ^b^**	**0.045 ^b^**	**0.091 ^a^**	**0.006**	**0.016**
Potential degradability (%)	88.3	81.7	81.0	79.4	1.85	0.160
Effective degradability (%)	64.6	63.5	64.4	67.7	1.34	0.954
Soybean meal						
*a (%)*	26.9	26.7	26.8	26.3	0.63	0.979
*b (%)*	71.2	69.5	72.6	71.3	1.02	0.784
*c* (h^−1^)	0.038	0.046	0.061	0.065	0.004	0.278
Potential degradability (%)	98.1	96.3	99.4	97.6	0.71	0.440
Effective degradability (%)	54.8	57.3	62.3	62.8	1.50	0.363
Barley straw						
*a* (%)	**12.8 ^b^**	**11.8 ^b^**	**11.9 ^b^**	**16.8 ^a^**	**0.30**	**0.001**
*b* (%)	30.9	27.6	27.0	27.3	1.74	0.901
*c* (h^−1^)	0.018	0.017	0.009	0.021	0.005	0.177
Potential degradability (%)	43.7	53.3	38.9	44.1	4.92	0.719
Effective degradability (%)	21.8	22.1	15.7	23.4	2.17	0.514
Sugar beet pulp						
*a (%)*	3.06	7.91	8.57	13.1	1.40	0.230
*b (%)*	96.3	92.2	87.9	84.3	2.32	0.436
*c* (h^−1^)	0.057	0.034	0.044	0.053	0.005	0.410
Potential degradability (%)	99.4	100.1	96.4	97.4	0.96	0.590
Effective degradability (%)	49.6	40.9	45.1	51.4	2.15	0.358

^1^ Concentrate without glycerin, with 20, 40 or 80 g of glycerin per kg of dry matter (0 G, 20 G, 40 G and 80 G, respectively). ^2^ SEM = standard error of the mean (n = 4 replicates per treatment). ^3^ Parameters of the exponential equation of Ørskov and McDonald [16]: $D\left(t\right)=a+b\;\left(1-e^{-ct}\right)$, where *D(t)* is the proportion (%) of the dry matter degraded at the time *t* (hours of incubation); *a* is the rapidly soluble fraction (%); *b* is the potentially degradable fraction (%), and *c* is the rate of degradation (h^−1^) of the fraction *b*. ^4^ The potential degradability values (%) were estimated as the sum of *a* + *b*. ^5^ The effective degradability values (%) were estimated as the sum of $a+\left[\frac{bc}{c+k}\right]$, where *a*, *b*, and *c* are the degradation constants estimated as described, and *k* is the fractional rate of passage assumed to be 0.06 h^−1^. ^6^ DDGS = corn distillers dried grains with solubles. ^7^ Row in boldface highlights that a statistically significant difference between treatments was found. ^a,b^ Means in the same row with different superscripts are significantly different (*p* < 0.05).
